# Multi-node inhibition targeting mTORC1, mTORC2 and PI3Kα potently inhibits the PI3K/AKT/mTOR pathway in endometrial and breast cancer models

**DOI:** 10.1038/s41416-025-03035-z

**Published:** 2025-05-13

**Authors:** Petros A. Tyrakis, Domen Kampjut, Georgina F. Steele, H. Jonathan G. Lindström, Deborah Chirnomas, Benjamin D. Hopkins, Marcus D. Goncalves, Siddhartha Mukherjee, Lewis C. Cantley, Oliver D. K. Maddocks

**Affiliations:** 1 Faeth Therapeutics R&D, CRUK Cambridge Institute, Li Ka Shing Centre, Robinson Way, Cambridge, UK; 2 Englander Institute for Precision Medicine, Meyer Cancer Center, Department of Physiology and Biophysics, Weill Cornell Medicine, New York, NY USA; 3 Department of Medicine, NYU Langone Health, New York, NY USA; 4 Department of Medicine, Columbia University Irving Cancer Research Center, Columbia University, New York, NY 10032 USA; 5 Dana-Farber Cancer Institute, Harvard Medical School, Boston, MA 02115 USA

**Keywords:** Cancer metabolism, Cancer therapy, Gynaecological cancer

## Abstract

**Background:**

While PI3K/AKT/mTOR signalling plays a critical role in cancer, targeting this pathway with single node inhibitors has limited efficacy due to several known factors such as pathway feedback reactivation, co-occurring pathway mutations, and systemic glucose dysregulation leading to hyperinsulinemia. While multi-node inhibition approaches have shown promising clinical efficacy, they require further mechanistic characterisation.

**Methods:**

Using models of endometrial and breast cancer, we evaluated the efficacy of a multi-node PI3K/AKT/mTOR pathway inhibitor approach utilising the dual mTORC1/mTORC2 inhibitor sapanisertib, PI3Kα inhibitor serabelisib and an insulin-supressing diet. Pathway signalling inhibition versus a range of single-node inhibitors was measured via S6, AKT and 4E-BP1 phosphorylation.

**Results:**

The serabelisib-sapanisertib combination more effectively suppressed PI3K/AKT/mTOR pathway signalling, particularly 4E-BP1, than single-node inhibitors, including alpelisib, capivasertib, inavolisib, everolimus and mutant-specific PI3K inhibitors RLY-2608 and STX-478. Serabelisib plus sapanisertib combined effectively with a range of other therapeutics, such as chemotherapies, hormone targeted therapies and CDK4/6 inhibitors. In xenograft models, sapanisertib, serabelisib plus paclitaxel/insulin supressing diet achieved complete inhibition of tumour growth/tumour regression.

**Conclusion:**

Multi-node PI3K/AKT/mTOR pathway inhibition with serabelisib, sapanisertib and ISD is highly effective in preclinical models of endometrial and breast cancer, supporting continued clinical development in these and other solid tumours.

## Introduction

**Fig. 1:**
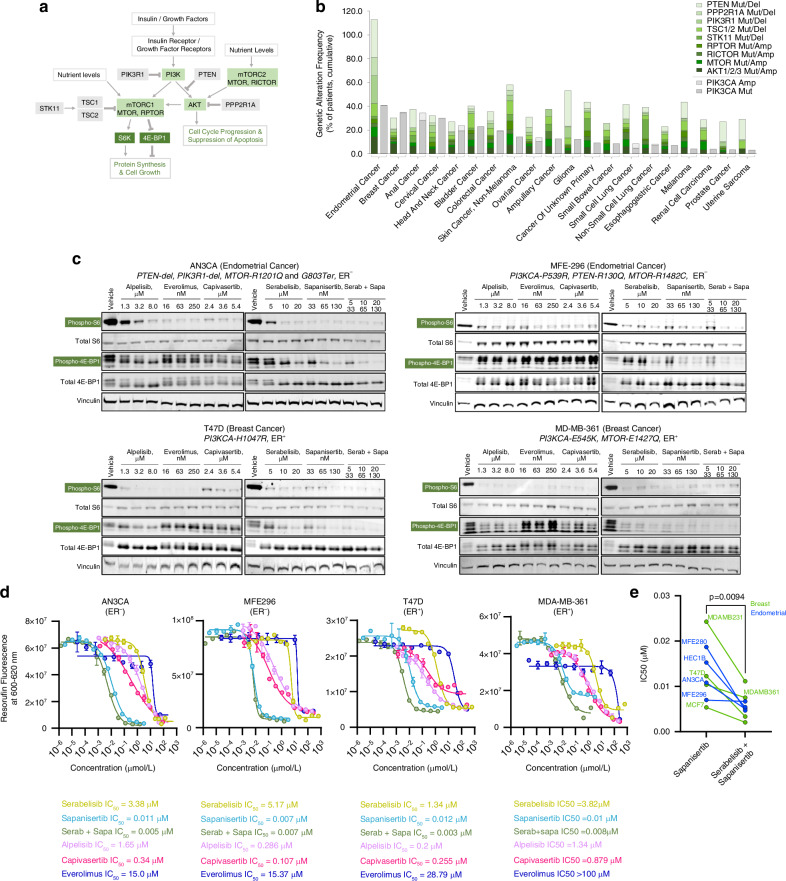
A multi-node targeting strategy utilizing sapanisertib and serabelisib achieves improved PI3K/AKT/mTOR pathway inhibition versus single node inhibitors. **a** Overview of PI3K/AKT/mTOR pathway. **b** Data sourced from MSK-IMPACT clinical sequencing cohort, *n* = 10,945 samples via cBioPortal (MSK, Nature Medicine 2017). Amp amplification, Mut mutation, Del homozygous deletion. The 20 tumour types with the highest cumulative mutation frequency are shown. Note that multiple mutations can occur within the same tumour sample, hence cumulative total can exceed 100%. **c** Western blots of PI3K/AKT/mTOR pathway output (phosS6-S235/236 and phos4EBP1-T37/46) in endometrial and breast cancer cell lines treated with the indicated inhibitors for 3–4 h followed by stimulation with 10 ng/ml insulin for 10 min. Drug concentrations were selected to represent clinical Cmaximum and Caverage for each drug and the mid-point of these. **d** Dose-response curves for indicated drugs at 72 h post-treatment. **e** IC50 values of sapanisertib compared with serabelisib + sapanisertib in breast and endometrial cancer cell lines; unpaired t-test. Error bars denote SD.

The PI3K/AKT/mTOR signalling pathway represents the major control hub for metabolic signalling in mammalian biology. This multi-node pathway senses nutrient and growth factor levels, and under favourable conditions signals to activate protein translation to support growth and proliferation, while simultaneously promoting cell cycle progression and inhibiting apoptosis (Fig. 1a). Consistent with its central role in controlling cellular growth and proliferation, mutations that activate PI3K/AKT/mTOR signalling are amongst the most common genetic alterations in cancer. While *PIK3CA* (the gene encoding PI3Kα) mutation is the most frequent PI3K-pathway related individual genetic alteration, broader pathway mutations cumulatively outweigh *PIK3CA* mutation in most solid tumours (Fig. 1b). Hormone sensitive cancers, particularly endometrial and breast cancer, have high PI3K pathway mutation rates. While approximately 80% of endometrial cancers carry a PI3K/AKT/mTOR pathway mutation [1, 2], surprisingly there are no approved PI3K/AKT/mTOR pathway targeted agents for this disease. Currently, single node inhibitors (SNIs) of the PI3K/AKT/mTOR pathway alpelisib and inavolisib (targeting PI3Kα) and capivasertib (targeting AKT) have received regulatory approval for breast cancer treatment, while everolimus (mTORC1 inhibitor) is approved for breast cancer and other tumour types. However, these SNIs have modest therapeutic benefit (e.g. everolimus in endometrial cancer [3]), due to known factors that limit efficacy which underlie the failure of SNI approaches to achieve regulatory approval for treatment of endometrial and other cancers.

The efficacy of single node inhibitors is limited by several factors which promote persistent or reactivated PI3K/AKT/mTOR signalling, including (i) pathway reactivation by feedback activation or bypass signalling, (ii) the presence of co-occurring pathway mutations beyond the targeted node [4], and (iii) systemic perturbations in glucose homeostasis that lead to hyperinsulinemia and pathway reactivation (summarised in Supplementary Fig. S1a). Pathway feedback reactivation rapidly occurs in response to SNIs as drugs targeting single pathway nodes alleviate negative feedback signalling. For example, upregulation of receptor tyrosine kinases such as HER3, that can re-engage pro-growth ERK signalling and abrogate drug activity [5–11]. Combinatorial strategies involving both PI3K/AKT/mTOR and MEK inhibition overcome this issue in preclinical models; however, the combination of PI3K/AKT/mTOR and MEK inhibitors is not tolerable in humans [12–15]. Resistance mutations that reactivate the PI3K/AKT/mTOR pathway occur in the context of PI3Kα inhibition, e.g., loss of PTEN/AKT activation in breast cancer patients treated with alpelisib or inavolisib, as well as further mutations in PI3Kα itself at progression [16, 17]. Resistance via pathway bypass can also occur to single node inhibition of PI3Kα, whereby mTOR activity is maintained via alternative effectors (i.e. effectors other than PI3Kα), such as PIM kinases [18].

Another well-known issue of PI3K/AKT SNIs is emergence of treatment-induced hyperglycaemia and hyperinsulinemia, which are propagated as ‘on-target, off tumour’ effects where blockade of PI3K/AKT/GLUT4 signalling prevents glucose uptake by peripheral tissues [19]. The resulting elevation of systemic insulin, the primary hormone which activates the PI3K-pathway (Fig. 1a), is a direct cause of treatment resistance but can be prevented by manipulation of diet. In several mouse models of cancer, an insulin supressing diet prevented glucose and insulin increases in response to PI3K inhibitors and significantly improved antitumor efficacy [19].

Another strategy to avoid hyperglycaemia is the development of mutant-specific PI3Kα inhibitors, which spare wild-type PI3Kα, e.g. RLY-2608 [20] and STX-478 [21]. However, mutant-specific inhibitors have the inherent limitations of SNIs (i.e. pathway feedback re-activation and/or resistance via mutations in *PTEN*/*AKT* [17]) and furthermore allow for continued pathway activity via remaining wild-type-PI3Kα retained by tumour cells. Hence, an alternative therapeutic paradigm is needed to address both the cell-autonomous mechanisms of resistance to SNIs, and the role systemic glucose and insulin play in reducing efficacy and increasing systemic toxicity of these agents.

A multi-node inhibitor (MNI) approach to blocking PI3K/AKT/mTOR signalling may overcome these issues. Recent clinical data for approaches targeting mTORC1, mTORC2 and PI3K (e.g. sapanisertib/serabelisib which target mTORC1, mTORC2 and PI3Kα, or pan-PI3K/mTOR inhibitor gedatolisib) have shown promising efficacy signals in cancer patients, suggesting that combined mTORC1/2 and PI3K inhibition may be the preferable MNI strategy [22–25]. For example, Starks et al., reported that the triplet combination of sapanisertib, serabelisib and paclitaxel leads to an objective response rate (ORR) of nearly 50% in a Phase 1b clinical trial of patients with advanced, treatment refractory, breast, endometrial, and ovarian cancers [22].

A key differentiating attribute of small molecule PI3K inhibitors is PI3K isoform selectivity, which contributes to immune cell suppression and relative activity in solid versus haematological cancers. Whereas cell types responsible for the development of solid tumours preferentially express PI3Kα, immune cells rely on PI3Kγ and PI3Kδ to control PI3K-pathway signalling and have low expression of PI3Kα [26]. Consequently, mutation rates for *PIK3CA* are at least 10-fold higher than *PIK3CB/PIK3CD/PIK3CG* in solid tumours such as breast and endometrial cancer (TCGA Research Network). Given the importance of immune effector cell function in combating cancer, and the increasing adoption of immunotherapies in solid tumour treatment, immune-sparing, α-specific PI3K inhibition is likely preferable to pan-PI3K isoform inhibition. For example, it has been shown that whereas α-specific PI3K inhibition with serabelisib has a minimal impact on B-cells and T-cells, pan-PI3K inhibition potently suppresses B-cell and T-cell expansion in vivo [27].

Here, we sought to explore the mechanism and efficacy of a multi-node PI3K/mTOR/AKT pathway inhibition approach in breast and endometrial cancer models. We find that the combination of serabelisib and sapanisertib more robustly inhibits PI3K/AKT/mTOR signalling than either agent alone or compared with other SNI and mutant-specific inhibitors. Importantly, the combination remained active in the context of multiple pathway mutations including *PTEN* loss. In addition, an insulin-suppressing diet improved the therapeutic response to these agents in vivo. The combination of sapanisertib and serabelisib showed anti-cancer efficacy when administered with a number of clinically relevant agents for breast and endometrial cancer, including palbociclib, fulvestrant, elacestrant, selinexor, carboplatin and paclitaxel.

## Results

### A multi-node inhibitor strategy utilizing sapanisertib and serabelisib achieves improved PI3K/AKT/mTOR pathway inhibition versus single node inhibitors alpelisib, capivasertib and everolimus

Endometrial and breast cancer cells harbouring a range of PI3K/AKT/mTOR pathway mutations were treated in vitro with concentrations of drug that reflect free-drug exposures in patients treated with these agents (i.e. clinically relevant concentrations of each agent, representing Caverage, Cmaximum and mid-point of these). Western blots show that while approved SNIs alpelisib, everolimus and capivasertib decreased S6 phosphorylation (signifying lower pro-growth signalling), they showed very limited ability to prevent 4E-BP1 phosphorylation (i.e. pro-growth phosphorylated-4E-BP1 signal was maintained) (Fig. 1c & Supplementary Fig. S1b). As a SNI, the α-specific PI3K inhibitor serabelisib was also able to inhibit S6 activation to a greater extent than 4E-BP1 activity – albeit serabelisib tended to inhibit 4E-BP1 phosphorylation to a greater extent than the α-specific PI3K inhibitor alpelisib. In contrast, sapanisertib, a second-generation mTOR inhibitor which targets both mTORC1 and mTORC2, achieved excellent inhibition of S6 phosphorylation and much improved inhibition of 4E-BP1 phosphorylation versus single-node inhibitors. By far the most complete pathway inhibition (i.e. simultaneously reduced signal for phosphorylated-S6 and phosphorylated 4E-BP1) was achieved by the combination of sapanisertib and serabelisib. This multi-node approach was able to dramatically inhibit PI3K/AKT/mTOR pathway activity even in cell lines with 2-3 PI3K/AKT/mTOR pathway mutations (Fig. 1c & Supplementary Fig. S1b).

### Sapanisertib and serabelisib potently inhibit cancer cell growth individually and in combination

Carboplatin and paclitaxel are mainstays in the early-line treatment of advanced/recurrent endometrial cancer. In endometrial cancer cell lines, carboplatin and paclitaxel mean IC50s were 265 μM and 18.5 nM respectively (Supplementary Fig. S1c). Among the PI3K/AKT/mTOR pathway inhibitors tested, sapanisertib showed lowest single agent IC50 (average = 13 nM, similar to paclitaxel) (Fig. 1d & Supplementary Fig. S1d). Furthermore, the combination of sapanisertib plus serabelisib, which had the greatest impact on 4E-BP1 phosphorylation, had an even lower mean IC50 of 5.5 nM, a significant improvement on sapanisertib alone (Fig. 1d, e & Supplementary Fig. S1d), and on average more potent than the other targeted and chemotherapies tested, and within the clinical exposure levels for these agents.

### Sapanisertib plus serabelisib achieves improved PI3K/AKT/mTOR pathway inhibition versus single node inhibitors inavolisib, RLY-2608 and STX-478

**Fig. 2:**
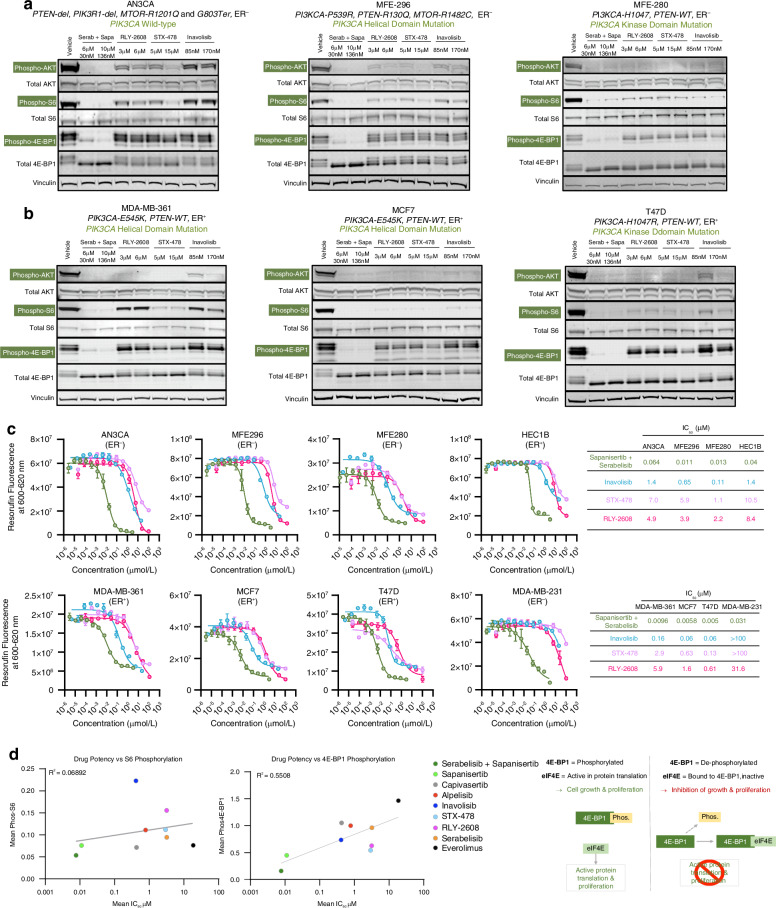
The combination of sapanisertib and serabelisib more completely inhibits PI3K/AKT/mTOR signalling than mutant-specific PI3Kα inhibitors. Western blots of PI3K/AKT/mTOR pathway activity (phosAKT-S473, phosS6-S235/236 and phos4E-BP1-T37/46) in endometrial (**a**) and breast (**b**) cancer cell lines, stimulated with 10 ng/ml insulin followed by treatment with the indicated inhibitors for 3–4 h. **c** Dose-response curves for serabelisib + sapanisertib against indicated mutant-specific PI3Kα inhibitors at 72 h post-treatment. **d** Correlation plot of PI3K/AKT/mTOR pathway inhibitors comparing the potency (IC50) of indicated drugs with the in vitro levels of phos-4E-BP1-T37/46 and phos-S6-S235/236 after 3–4 hours treatment with indicated drugs at the average free-drug plasma concentration (Caverage) achieved in humans/in vivo models; data are means for breast and endometrial cancer cell lines used in this study. A diagram depicting action of 4E-BP1 and phoph4E-BP1 on eIF4E is also shown in panel (**d**). Error bars denoted SD.

Inavolisib (α-specific PI3K inhibitor/degrader), RLY-2608 and STX-478 (mutant specific PI3K inhibitors) are SNIs of the PI3K/AKT/mTOR pathway currently in clinical development. In similarity to clinically approved SNIs (i.e. alpelisib, everolimus and capivasertib) inavolisib, RLY-2608 and STX-478 showed only limited ability to inhibit PI3K/AKT/mTOR pathway signalling in breast and endometrial cancer cells (Fig. 2a, b). At clinically relevant concentrations these SNIs generally achieved variable degrees of inhibition of S6 and AKT phosphorylation but had little impact on 4E-BP1-phosphorylation. In contrast, the MNI combination of sapanisertib plus serabelisib generally showed a greater inhibition of S6 and AKT phosphorylation and substantially greater inhibition of 4E-BP1 phosphorylation in endometrial and breast cancer cells. Notably the MNI approach outperformed the SNIs independently of whether the cells harboured wild-type *PIK3CA*, helical domain mutations of *PIK3CA*, kinase domain mutations of *PIK3CA*, or *PTEN* loss-of-function mutations. Also of note, despite the substantial cross-talk between Estrogen Receptor (ER) signalling and the PI3K/AKT/mTOR pathway, ER status did not impact the potency of serabelisib and sapanisertib in breast and endometrial cancer cell lines.

Compared to mutant-specific PI3Kα inhibitors (RLY-2608 & STX-478) and PI3Kα inhibitor/degrader inavolisib, the sapanisertib plus serabelisib combination had a substantially lower mean IC50 i.e., 32.0 nM versus 0.89 μM (inavolisib), 6.13 μM (STX-478), 4.85 μM (RLY-2608) (Fig. 2c). A very similar pattern of results was observed in a range of breast cancer cell lines. Mean IC50 of sapanisertib alone (12.9 nM) or in combination with serabelisib (6 nM) was superior to other clinically relevant agents such as paclitaxel (90.8 nM), fulvestrant (437 nM for ER+ cell lines), alpelisib (5.5 μM), capivasertib (22.3 μM), everolimus (60.9 μM) and CDK4/6 inhibitors abemaciclib (30.8 μM), palbociclib (17.2 μM) & ribociclib (51.2 μM) (Fig. 2c & Supplementary Fig. S2a). Compared to mutant-specific PI3Kα inhibitors and inavolisib, which had micromolar IC50, sapanisertib/serabelisib also had a substantially lower (i.e. nanomolar) IC50 (Fig. 2c).

#### Inhibition of 4E-BP1 (but not S6) phosphorylation correlates with drug potency

Given the marked difference between MNI and SNI approaches with respect to suppression of 4E-BP1 phosphorylation, we hypothesised that inhibition of 4E-BP1 phosphorylation, rather than inhibition of S6 phosphorylation, would be correlated with dose-response. Notably, there was a strong correlation between drug potency (IC50) and inhibition of 4E-BP1 phosphorylation, whereas inhibition of S6 phosphorylation did not correlate with drug potency (Fig. 2d).

### Sapanisertib plus serabelisib inhibits cancer cell growth in combination with a range of targeted and chemotherapeutic anticancer agents

**Fig. 3:**
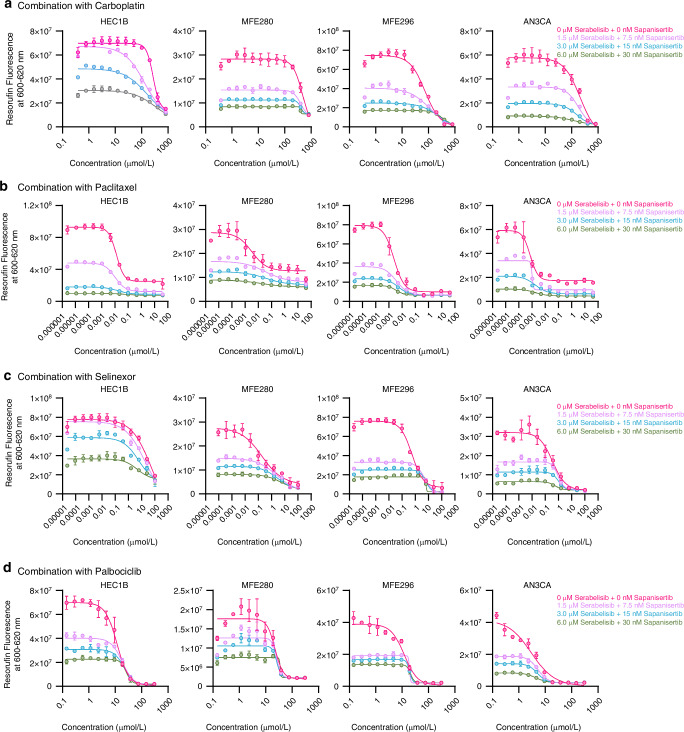
Sapanisertib and serabelisib enhance endometrial cancer cell growth suppression in combination with a range of targeted and chemotherapeutic anticancer agents. **a** Dose-response curves for carboplatin with different concentrations of serabelisib plus sapanisertib at 72 h post-treatment in endometrial cancer cell lines. **b** Dose-response curves for paclitaxel with different concentrations of serabelisib plus sapanisertib at 72 h post-treatment in endometrial cancer cell lines. **c** Dose-response curves for selinexor with different concentrations of serabelisib plus sapanisertib at 72 h post-treatment in endometrial cancer cell lines. **d** Dose-response curves for palbociclib with different concentrations of serabelisib plus sapanisertib at 72 h post-treatment in endometrial cancer cell lines. Error bars denote SD.

There is an increasing reliance on therapeutic combinations in cancer treatment. We sought to better understand the ability of sapanisertib plus serabelisib to combine with agents with different mechanisms of action in a range of endometrial (AN3CA, MFE296, MFE280 & HEC1B) and breast (MDA-MB-361, MCF7, T47D & MDA-MB-231) cancer cell lines. Increasing clinically relevant doses of sapanisertib and serabelisib produced both down- and left-shifted growth inhibition curves for carboplatin, paclitaxel, selinexor (selective inhibitor of nuclear export being developed for *TP53* wild-type endometrial cancer) and palbociclib (CDK4/6 inhibitor) in endometrial cancer lines (Fig. 3a–d). This trend was consistent across all four endometrial cancer cell lines tested, and there was no evidence of agent-to-agent antagonism (i.e. to right/upward-shift of curves). Overall, these data support the ability to combine sapanisertib plus serabelisib with a range of established/emerging therapeutics for endometrial cancer. A very similar set of results was obtained with breast cancer cell lines, where inhibition curves were down/left-shifted in combination with paclitaxel, palbociclib, selinexor, fulvestrant and elacestrant (Supplementary Fig. S3a–d).

### Taxane resistance activates the PI3K/AKT/mTOR pathway, and taxane resistant cells retain sensitivity to sapanisertib plus serabelisib

**Fig. 4:**
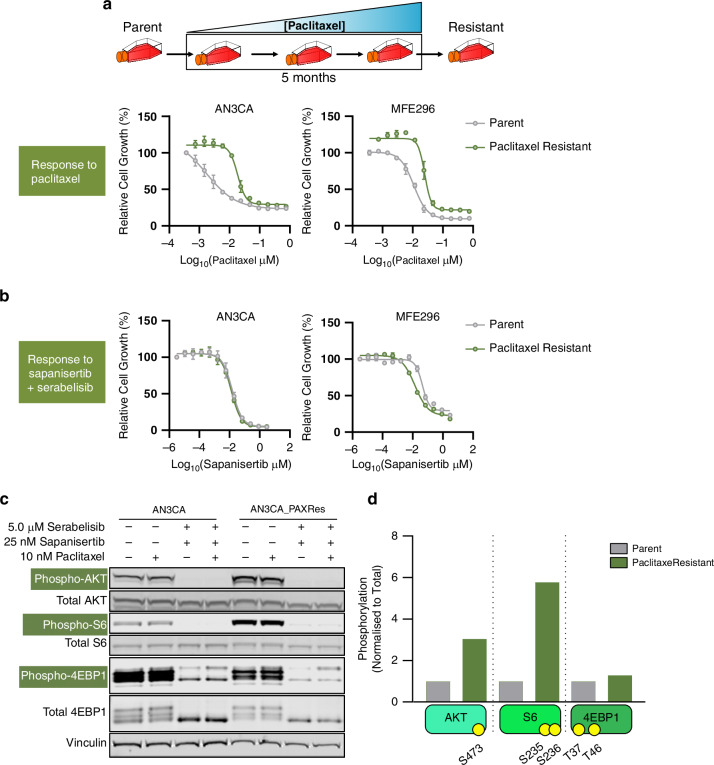
Taxane resistance activates the PI3K/AKT/mTOR pathway, and taxane resistant cells retain sensitivity to sapanisertib plus serabelisib. **a** Generation of paclitaxel resistant cell lines, with dose-response curves of paclitaxel in parental and paclitaxel resistant cell lines at 72 h post-treatment. **b** Dose-response curves of sapanisertib plus serabelisib in parental and paclitaxel resistant cell lines at 72 h post-treatment. **c** Western blots of PI3K/AKT/mTOR pathway activity (phosAKT-S473, phosS6-S235/236 and phos4EBP1-T37/46) in AN3CA parental or paclitaxel resistant cell lines, stimulated with 10 ng/ml insulin followed by treatment with the indicated inhibitors for 3–4 h. **d** Quantification of the indicated phospho-AKT/S6/4EBP1 levels in vehicle-treated AN3CA parental or paclitaxel resistant samples. Error bars denote SD.

Given the positive in vitro combination data in the present study, and efficacy signal seen in a Phase 1b clinical trial combining sapanisertib, serabelisib and paclitaxel [22], in which several patients who had received paclitaxel in prior lines of therapy had partial or complete clinical responses, we sought to further explore the relationship between paclitaxel treatment and the PI3K/AKT/mTOR pathway. After five months exposure to increasing concentrations of paclitaxel the endometrial cancer cell lines AN3CA and MFE296 acquired paclitaxel resistance, as demonstrated by substantial right-shift in the paclitaxel growth inhibition curves (Fig. 4a). Despite acquiring taxane resistance, the cells either remained sensitive (AN3CA), or displayed increased sensitivity (left-shifted curve, MFE296 cells) to sapanisertib plus serabelisib (Fig. 4b).

Analysis of PI3K/AKT/mTOR pathway signalling revealed that taxane-resistant endometrial cancer cells had increased pathway activity: taxane-resistant AN3CA cells displayed 6-fold higher phosphorylated-S6 and 3-fold elevation in phosphorylated-AKT (Fig. 4c, d). The effect was more modest in MFE296 cells, which showed a 2-fold increase in phosphorylated-S6 (Supplementary Fig. S4a, b). Notably, despite the increases in phosphorylation of pathway effectors, the sapanisertib plus serabelisib combination was able to inhibit signalling equally as well in parental cells or taxane resistant cells (Fig. 4c, d, Supplementary Fig. S4a, b). Taken together these data suggest that taxane resistance in endometrial cancer cells involves upregulated PI3K/AKT/mTOR pathway activity, however this increase does not reduce – but rather potentially increases – sensitivity to sapanisertib plus serabelisib.

To replicate a clinically relevant dosing regimen for sapanisertib, serabelisib and paclitaxel in vitro, we conducted an experiment where drugs were given sequentially and for a discrete time period. I.e. paclitaxel was given on day 1 (and removed after 24 h), and sapanisertib plus serabelisib were given on days 2, 3 and 4. As with other in vitro experiments, clinically relevant concentrations of sapanisertib and serabelisib were used. Paclitaxel was used at low doses to allow for the scale of sensitization to be revealed. In the four endometrial cancer cell lines tested, the sapanisertib, serabelisib and paclitaxel combination caused substantial growth inhibition and, as expected, outperformed single drug and double-drug combinations (Supplementary Fig. S4c).

### Sapanisertib and serabelisib in combination with paclitaxel strongly suppress tumour growth of in vivo models of endometrial and breast cancer, which is further enhanced by an insulin-suppressing diet

**Fig. 5:**
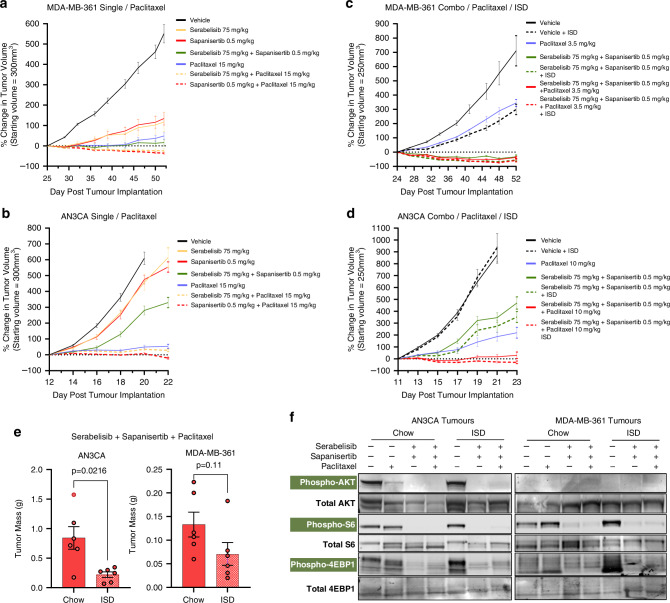
Sapanisertib and serabelisib in combination with paclitaxel strongly suppress in vivo tumour growth of endometrial and breast cancer models, which is further enhanced by an insulin-suppressing diet. **a** Efficacy of sapanisertib (0.5 mg/kg), serabelisib (75 mg/kg), paclitaxel (15 mg/kg) and their combinations in MDA-MB-361 xenografts. Drugs were initiated when tumour volume reached 300 mm^3^; *n* = 7–8 mice per group. **b** Efficacy of sapanisertib (0.5 mg/kg), serabelisib (75 mg/kg), paclitaxel (15 mg/kg) and their combinations in AN3CA xenografts. Drugs were initiated when tumour volume reached 300 mm^3^; *n* = 8 mice per group. **c** Efficacy of sapanisertib (0.5 mg/kg), serabelisib (75 mg/kg), paclitaxel (3.5 mg/kg) and their combinations, with and without an insulin-suppressing diet (ISD) in MDA-MB-361 xenografts. Drugs were initiated when tumour volume reached 250 mm^3^, *n* = 5–8 mice per group. **d** Efficacy of sapanisertib (0.5 mg/kg), serabelisib (75 mg/kg), paclitaxel (10 mg/kg) and their combinations, with and without ISD in AN3CA xenografts. Drugs were initiated when tumour volume reached 250 mm^3^, *n* = 6–8 mice per group. **e** AN3CA and MDA-MB-361 tumours were randomly harvested from chow and ISD groups treated with sapanisertib, sapanisertib and paclitaxel at study endpoint; *n* = 6 per group. Unpaired t-test with Welch’s correction. **f** Representative Western blots of PI3K-AKT-mTOR pathway activity (phosAKT-S473, phosS6-S235/236 and phos4EBP1-T37/46) in AN3CA and MDA-MB-361 tumours from indicated groups, 1 h post drug dosing; *n* = 3 tumours per group were randomly harvested and processed for Western blot. Error bars denote SEM.

Given the in vitro mechanistic data demonstrating cooperativity between sapanisertib, serabelisib and paclitaxel, we conducted in vivo efficacy experiments using endometrial and breast cancer xenografts. In the first set of experiments, we used single and double drug combinations (Fig. 5a, b), in the second set of experiments we used double and triple drug combinations with or without an insulin suppressing diet (Fig. 5c, d). Doses used represent the mouse equivalent area under the curve (AUC) exposure for 200 mg serabelisib and 3 mg sapanisertib in humans (RP2D for sapanisertib plus serabelisib combination [22]), administered in the clinically relevant dosing schedule (paclitaxel once per week on day 1, sapanisertib and serabelisib (PO) 3 days per week on days 2, 3 and 4). Drug treatments were initiated once tumours were well-established and growing rapidly (250–300 mm^3^).

As single agents, sapanisertib and serabelisib showed substantial inhibition of tumour growth in MDA-MB-361 tumours, and activity in AN3CA tumours. This result correlates with Western blot data (Fig. 1c) showing 4E-BP1 inhibition by sapanisertib and serabelisib was achieved in both cell lines but was more complete in MDA-MB-361 cells. In combination, sapanisertib plus serabelisib showed increased efficacy in both models versus single agents, with near complete tumour growth inhibition in MDA-MB-361 cells (Fig. 5a, b). Both models were relatively sensitive to paclitaxel (at 15 mg/kg dose), which caused substantial tumour growth inhibition as monotherapy. Notably, paclitaxel monotherapy was less effective than sapanisertib and serabelisib combination for MDA-MB-361 cells. Doublets of sapanisertib + paclitaxel (15 mg/kg) or serabelisib + paclitaxel (15 mg/kg) showed very robust tumour growth inhibition in both models. In AN3CA cells the sapanisertib + paclitaxel combination was especially effective, causing tumour regression. In MDA-MB-361 cells both doublet drug combinations produced tumour regression.

Given the potential for sapanisertib and serabelisib to sensitize tumours to paclitaxel we sought to assess the triplet combination (sapanisertib, serabelisib, paclitaxel). As the doublet combinations (i.e. sapanisertib plus paclitaxel or serabelisib plus paclitaxel) were highly efficacious (Fig. 5a, b), we selected reduced doses of the taxane (10 mg/kg in AN3CA and 3.5 mg/kg in MDA-MB-361) for the triplet experiments to allow for the scale of sensitization to be revealed (Fig. 5c, d). In both xenograft models the drug triplet caused profound tumour growth inhibition, with tumour regression seen in both models, especially MDA-MB-361. This degree of tumour growth inhibition was particularly notable given the lowered doses of paclitaxel and the starting tumour volume (250 mm^3^).

We next sought to establish the impact on efficacy of adding ISD to the sapanisertib, serabelisib and paclitaxel regimen. Notably ISD alone had a major impact on MDA-MB-361 xenografts, inhibiting tumour growth to a greater extent than 3.5 mg/kg paclitaxel (Fig. 5c). When added to the sapanisertib plus serabelisib doublet, or sapanisertib, serabelisib and paclitaxel triplet, ISD enhanced anti-tumour efficacy (Fig. 5c, d). When added to the sapanisertib plus serabelisib doublet, or sapanisertib, serabelisib and paclitaxel triplet in AN3CA xenografts, ISD enhanced anti-tumour efficacy, resulting in lower tumour mass at endpoint (Fig. 5e). The drug-drug/drug-diet combinations were well-tolerated (Supplementary Fig. S5a–d), while some weight loss was seen in ISD groups initially this tended to stabilize over the course of the study. As seen with in vitro experiments, tumours treated with sapanisertib plus serabelisib showed a substantial decrease in AKT, S6 and 4E-BP1 phosphorylation (Fig. 5f).

Taken together these data show that sapanisertib plus serabelisib, with the optional addition of ISD, represents a comprehensive multi-nodal approach for the effective inhibition of PI3K/AKT/mTOR signalling in tumours in vivo (Supplementary Fig. S5e). Furthermore, combination of multi-node PI3K/AKT/mTOR inhibition with paclitaxel – even at paclitaxel doses with relatively modest monotherapy efficacy – produced profound tumour growth inhibition and tumour regression in preclinical models of endometrial and breast cancer.

## Discussion

While much attention has been given to the fact that *PIK3CA* (encoding PI3Kα) is a frequently mutated gene in cancer, it is striking that mutations in the broader PI3K/AKT/mTOR pathway significantly outweigh *PIK3CA* mutations in solid tumours. Attempts to drug the PI3K/AKT/mTOR pathway have given substantial effort to developing PI3K inhibitors; irrespective of whether such inhibitors target wild-type or mutant PI3K, this single-node approach has significant limitations, i.e. substantially reducing the eligible patient population, delivering incomplete pathway inhibition, and treatment resistance driven by mutations beyond the targeted node. Recent clinical data for multi-node PI3K/mTOR/AKT pathway inhibition approaches has shown promising efficacy signals in cancer patients [22–25]. Whilst previous attempts to develop multi-node PI3K/AKT/mTOR pathway inhibitors comprising single small molecules (i.e. pan-isoform-PI3K-mTOR inhibitors) have shown promising efficacy, they have generally been limited by toxicity due in part to lack of target selectivity. For this reason, we sought to explore a combination of molecules with improved target selectivity, hitting core nodes throughout the pathway (mTORC1, mTORC2 and PI3Kα) while avoiding inhibition of PI3K signalling in immune cells (controlled by PI3Kγ and PI3Kδ isoforms).

Regarding PI3K/AKT/mTOR pathway inhibition, we found that targeting mTORC1, mTORC2 and PI3Kα with sapanisertib and serabelisib dramatically outperformed single node inhibitors, including mutant-specific inhibitors (STX-478 and RLY-2608), capivasertib, alpelisib and inavolisib. In relation to cancer cell growth inhibition, the superior potency of sapanisertib/sapanisertib plus serabelisib versus other inhibitors correlated with the ability to inhibit 4E-BP1 (rather than S6) phosphorylation at clinically relevant concentrations. This observation is consistent with the specific roles that S6 and 4E-BP1 are reported to play as effectors of PI3K/AKT/mTOR pathway signalling; whereas S6 tends to control cell size [28], 4E-BP1 is linked to cell proliferation [29]. As 4E-BP1 controls the rate-limiting step of cap-dependent translation, effective pharmacological targeting of PI3K/AKT/mTOR pathway signalling necessitates inhibition of 4E-BP1 function, which is also highlighted by the prognostic significance of 4E-BP1 phosphorylation in multiple cancers [30].

Sapanisertib and serabelisib combined positively with a range of other targeted and chemotherapeutic anticancer agents. We also found that paclitaxel-resistant cells had activated PI3K/AKT/mTOR pathway signalling, while having retained or enhanced sensitivity to multi-node inhibition. This observation complements previous work linking taxane resistance to upregulated PI3K/AKT/mTOR pathway activity [31], and work showing that PI3Kα inhibitor alpelisib sensitises gastric cancer models to paclitaxel [32]. Furthermore, the present study is complementary to work showing that sapanisertib combines synergistically with serabelisib or paclitaxel in models of bladder cancer [33], and is in-line with the positive clinical responses in the Phase 1b study in patients receiving the sapanisertib, serabelisib, paclitaxel triplet who had previously progressed on taxane treatment [22].

Besides achieving relatively modest efficacy, single node PI3K/AKT/mTOR pathway inhibitors have been benighted by on-target/off-tumour toxicity, particularly hyperglycaemia. This raises the potential concern that multi-node inhibition, which has improved on-target PI3K-pathway inhibition, will have a further increased risk of toxicity. In the present study, low doses of the sapanisertib-serabelisib combination achieved more complete PI3K/AKT/mTOR pathway inhibition than the highest doses of single node inhibitors. Notably, the clinical recommended Phase 2 combined doses of sapanisertib (3 mg) and serabelisib (200 mg) are 3-fold and 4.5-fold lower respectively than recommended Phase 2 monotherapy doses. Taken together, this suggests that MNI allows lower doses of drug to be used, while achieving more complete inhibition of pro-growth signalling in tumour tissue, which provides a greater therapeutic window than SNIs. The relatively low rates of hyperglycaemia seen in cancer patients treated with sapanisertib, serabelisib and paclitaxel (i.e. less than half the rates seen in SNI clinical trials [22]) supports this theory.

In addition to optimising drug selectivity and dose to enhance efficacy and safety, the control of systemic metabolism by dietary manipulation is shown to be a highly effective strategy for improving safety and efficacy of PI3K inhibitors in preclinical cancer models [19]. In the present study we found than an insulin supressing diet improved the antitumour efficacy of multi-node pathway inhibition. Beyond controlling systemic insulin and glucose levels, diet has additional potential mechanisms to promote antitumour efficacy; multiple studies have now shown that elevation of ketones such as beta-hydroxybutyrate (BHB) can have antitumour properties independent of insulin [34–36]. Taken together these preclinical data suggest that ISD is a viable option to improve the safety and efficacy of PI3K/AKT/mTOR pathway inhibiting drugs.

Our observation that taxane resistance elevates PI3K-pathway signalling in cancer cells complements the links between taxane sensitisation/resistance and the PI3K/AKT/mTOR pathway [37]. Given the central importance of PI3K/AKT/mTOR signalling in controlling protein translation and supressing apoptosis, it has the potential to be involved in the resistance mechanism to numerous anticancer therapies. The ability of cancer cells to activate and upregulate any program of drug resistance which involves increased expression of proteins (e.g. drug efflux transporters) necessitates active protein translation and therefore PI3K/AKT/mTOR pathway engagement. This is reflected in evidence that PI3K/mTOR/AKT signalling is implicated in resistance to CDK4/6 inhibitors, endocrine therapies, HER2-targeted therapy, chemotherapy, PARP inhibitors, radiation and immunotherapy in breast cancer (reviewed by Dong et al. [38]). In the present study, sapanisertib plus serabelisib showed a consistent ability to combine positively with other agents, including CDK4/6 inhibitors, selinexor, fulvestrant, paclitaxel and carboplatin.

Until recently, sapanisertib and serabelisib were themselves primarily developed as single-node PI3K/AKT/mTOR pathway inhibitors and are well-tolerated in humans [39–42]. As single agents, or in combination with other agents (such as paclitaxel), these drugs have shown signals of clinical efficacy, albeit consistent with the more modest activity of single node inhibitors [41, 43–49]. Overall, the preclinical data presented in this study, alongside recent clinical data, suggests that sapanisertib plus serabelisib represents a potentially viable MNI strategy compatible with chemo/targeted therapies for a range of solid tumours. Furthermore, concurrent utilisation of an insulin supressing diet offers potential to control systemic glucose and insulin for improved drug safety and efficacy.

## Methods

### Drugs and compounds

The following compounds, >95% purity, were used in this study (supplier information in parentheses): serabelisib (Carbogen), sapanisertib (Cayman Chemical, 11811), paclitaxel (Apex Bio, A4393), carboplatin (Sigma, BP711), alpelisib (Cayman Chemical, 16986), capivasertib (Biorbyt, ORB1307109), everolimus (Cayman Chemical, 11597), inavolisib (Cayman Chemical, 37576), RLY-2608 (Medchem Express, HY-153306), STX-478 (Medchem Express, HY-156681) fulvestrant (Cayman Chemical, 10011269), elacestrant hydrochloride (Cayman Chemical 37298). Stock solutions of each compound were made in the appropriate vehicle and stored at −20 °C.

### Cell culture

The following cell lines were used, the supplier from which each cell line was purchased is indicated in parentheses: AN3CA (ATCC), HEC1B (ATCC), MFE296 (ECACC), MFE280 (ECACC), MCF7 (ECACC), T47D (ECACC), MDA-MB-361 (ATCC), MDA-MB-361-Luc (JCRB), MDA-MB-231Cas9 (BPS Biosciences). AN3CA, HEC1B, MFE296, MFE280, MCF7, T47D, MDA-MB-231 cells were cultured in high glucose DMEM with 10% FBS + 2 mM glutamine. MDA-MD-361 cells were cultured in RPMI with 20% FBS + 2 mM glutamine. All cell lines were authenticated by STR genotyping and were free from mycoplasma, as assessed by Mycoprobe (R&D Systems). Cells were grown in 21% O_2_, 5% CO_2_ at 37 °C.

### Cell-based dose-response curves

Cells were seeded in 96-well plates. 24 h later, dilution series of compounds indicated in each figure were added to the appropriate wells. Cells were then cultured for a further 72 h followed by the addition of resazurin for 3–4 h. Alive cells were quantified by measuring the fluorescence of resorufin (reduced resazurin) at 600–620 nm. For the experiment with time-specific drug exposures (Supplementary Fig. S4c) with serabelisib, sapanisertib and paclitaxel, cells were seeded in 96-well plates on day 0. 24 h later (day 1), cells were treated with 10 nM paclitaxel or vehicle (DMSO). After 24 h (day 2), media was replaced, followed by the addition of either vehicle (DMSO), 5.9 µM serabelisib, 30 nM sapanisertib or 5.9 µM serabelisib +30 nM sapanisertib until day 4. On day 4, resazurin was added for 4 h and live cells were quantified by measuring the fluorescence of resorufin (reduced resazurin) at 600–620 nm.

### Immunoblotting

Cells were seeded in 10 cm dishes. The following day, media was replaced with serum-free media overnight (10–12 h). Depending on the experiment, insulin was added before or after drug treatment, as indicated in the figure legends. Cells were then washed rapidly with ice-cold PBS and harvested on ice by scraping. Cell pellets were flash frozen. Protein was extracted with RIPA buffer + HALT protease and phosphatase inhibitor (Thermo Fisher). Protein was quantified with the Bradford assay (Thermo Fisher) and 20 µg of each protein sample was subjected to SDS-PAGE. For immunoblot on tumour tissue, tumours were homogenised by grinding with a pestle and mortar on dry ice to a fine powder. Protein was then extracted and processed as above. Immunoblot analysis was performed using primary antibodies against the following targets: panAKT (Cell Signalling Technologies, 2920), phosAKT-Ser473 (Cell Signalling Technologies, 4058), S6 (Cell Signalling Technologies, 2317), phosS6-Ser235/Ser236 (Cell Signalling Technologies, 2211), 4E-BP1 (ProteinTech, 60246-1-Ig), phos4E-BP1-T37/46 (Cell Signalling Technologies, 2855), and vinculin (Cell Signalling Technologies, 13901). Secondary antibodies goat anti-rabbit-800 (926-32211) and donkey anti-mouse-680RD (926-68072) from Li-core were used. Membranes were visualised on a Licor Odyssey CLx imaging system. Quantification of immunoblots was performed using Licor Image Studio software.

### Generation of paclitaxel resistant endometrial cancer cell lines

AN3CA and MFE296 were cultured in gradually increasing doses of paclitaxel, starting from 2 nM, until colonies formed over a period of 3–4 months. Paclitaxel resistant cultures became established over the following month and were then maintained in 10 nM (AN3CA) or 18 nM (MFE296) paclitaxel continuously. Paclitaxel resistance was confirmed by performing dose-response curves against parental lines.

### Tumour xenografts

Female BALB/c nude mice 6–8 weeks of age were used for all in vivo experiments. Prior to implantation, AN3CA cells were cultured in vitro in EMEM medium supplemented with 10% heat inactivated FBS and MDA-MB-361 cells in L-15 medium supplemented with 20% heat inactivated FBS. Cells were in the exponential growth phase when harvested for implantation. Tumour cells were injected subcutaneously on the right flank of female BALB/c nude mice. For AN3CA, 5 million cells were implanted in 0.1 ml of growth medium; for MDA-MB-361, 10 million cells were implanted in 0.1 ml of 1:1 growth medium : Matrigel. The day before implantation of MDA-MB-361 cells, mice were given 40 μg of subcutaneous oestradiol benzoate and then twice per week thereafter for the duration of the experiment. Groups of 7–8 mice with tumours of the requisite size were recruited into treatment arms, such that the average tumour size was equal between groups at the start of the experiment. Mice that lost condition due to bladder calculi resulting from oestradiol injections were excluded from the study, as were mice that lost condition due to rolling in the ISD, which unlike regular chow pellets is a paste. Serabelisib was formulated in 5% DMSO, 5% Tween 80, 40% PEG300, 50% water, pH = 3.0 and administered by oral gavage 3 times per week at the doses indicated in each figure. Sapanisertib was formulated in 0.05% Tween 80 and 0.5% CMC in water and administered by oral gavage 3 times per week at the doses indicated in each figure. Paclitaxel was formulated in normal saline and administered intravenously via the tail vein, once per week at the indicated doses in each figure. Body weight of all mice was measured two to three times per week. Tumour dimensions were measured regularly with a calliper and tumour volume was estimated using the formula: TV = a × b^2^/2, where “a” and “b” are long and short diameters of a tumour, respectively. Skilled CRO staff, who were not blinded to the group allocations, but were blinded to the aims of the study, administered the diet(s) and drugs, as well as performed tumour and body weight measurements. Unless otherwise stated, mice were fed standard chow for the duration of the experiment. For pharmaco-dietary experiments, a high fat insulin-suppressing diet was used (HF93.5, Dyets, % w/w of macronutrients; protein = 8.9%, total carbohydrates (digestible and non-digestible) = 12.8%, fat = 78.4%), the control group received normal chow diet (Beijing Keao Xielie Feed Co. Ltd.). In the relevant groups, ISD was introduced 2 days before drug treatment was initiated.

### Statistical analyses

Statistical analyses were performed in GraphPad Prism 10 software. Comparisons were assessed using an unpaired two-tailed Student’s *t* test, with Welch’s correction when appropriate. Error bars are shown as s.d. unless otherwise indicated in figure legends and all experiments were performed at least twice. Sample sizes were chosen based on previous experience of in vitro and in vivo experiments.

## Supplementary information

Supplementary Figures

Author checklist

## Data Availability

The data that supports the findings of this study are available from the corresponding author upon reasonable request.
