# Supplementary material for: Multi-node inhibition targeting mTORC1, mTORC2 and PI3Kα potently inhibits the PI3K/AKT/mTOR pathway in endometrial and breast cancer models
Source: Br J Cancer. 2025 May 13;133(2):144–54. doi: 10.1038/s41416-025-03035-z (PMC12304390; doi:10.1038/s41416-025-03035-z)
Supplement: Supplementary file 1 — Supplementary Figures [file 41416_2025_3035_MOESM1_ESM.pdf]

Supplementary Figure 1

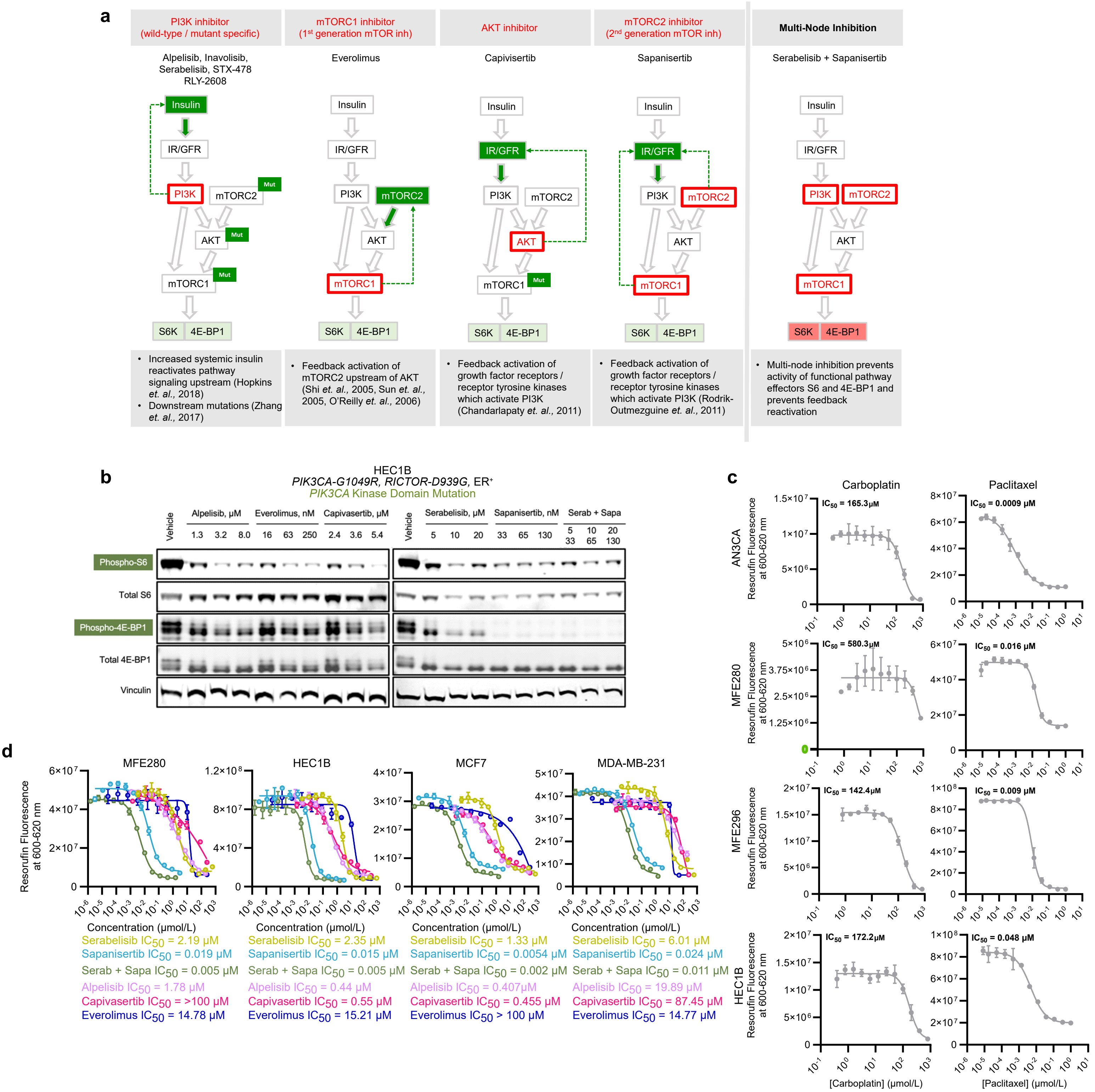

Supplementary Figure 1: A multi-node targeting strategy utilizing sapanisertib and serabelisib achieves improved PI3K/AKT/mTOR pathway inhibition versus single node inhibitors. a. Schematic diagram illustrating known mechanisms of pathway reactivation in response to drugs targeting the PI3K/AKT/mTOR pathway. **b.** Western blot of PI3K/AKT/mTOR pathway output (phosS6-S235/236 and phos4EBP1-T37/46) in HEC1B endometrial cancer cell line treated with the indicated inhibitors for 3-4 hours followed by stimulation with 10 ng/ml insulin for 10 minutes. **c.** Dose-response curves for indicated drugs at 72 hours post-treatment. **d.** Dose response curves for indicated drugs at 72 hours post-treatment. Error bars denote SD.

Supplementary Figure 2

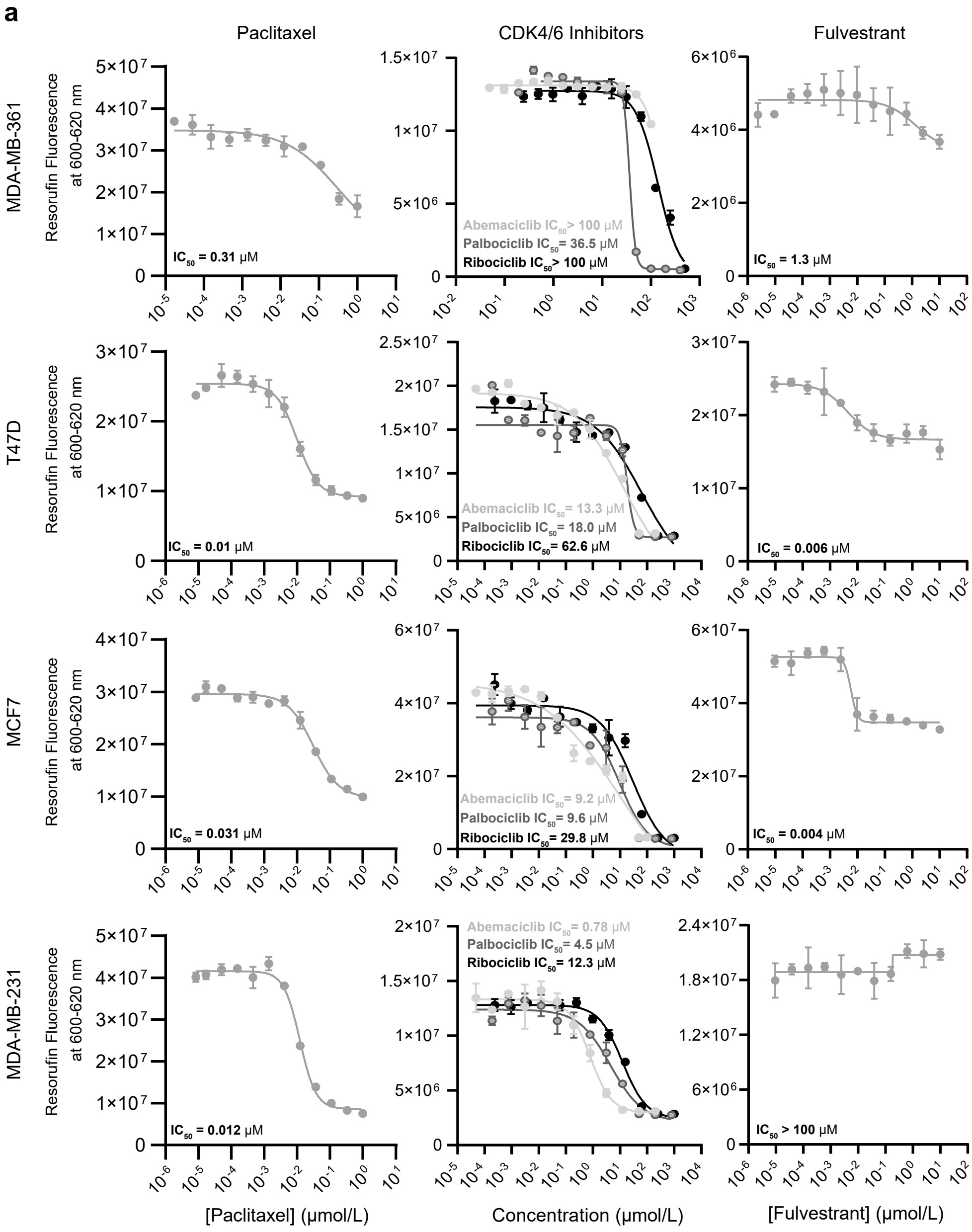

Supplementary Figure 2: Dose-response curves for standard of care agents in breast cancer cell lines. **a.** Dose-response curves for indicated standard of care drugs at 72 hours post-treatment. Error bars denote SD.

Supplementary Figure 3

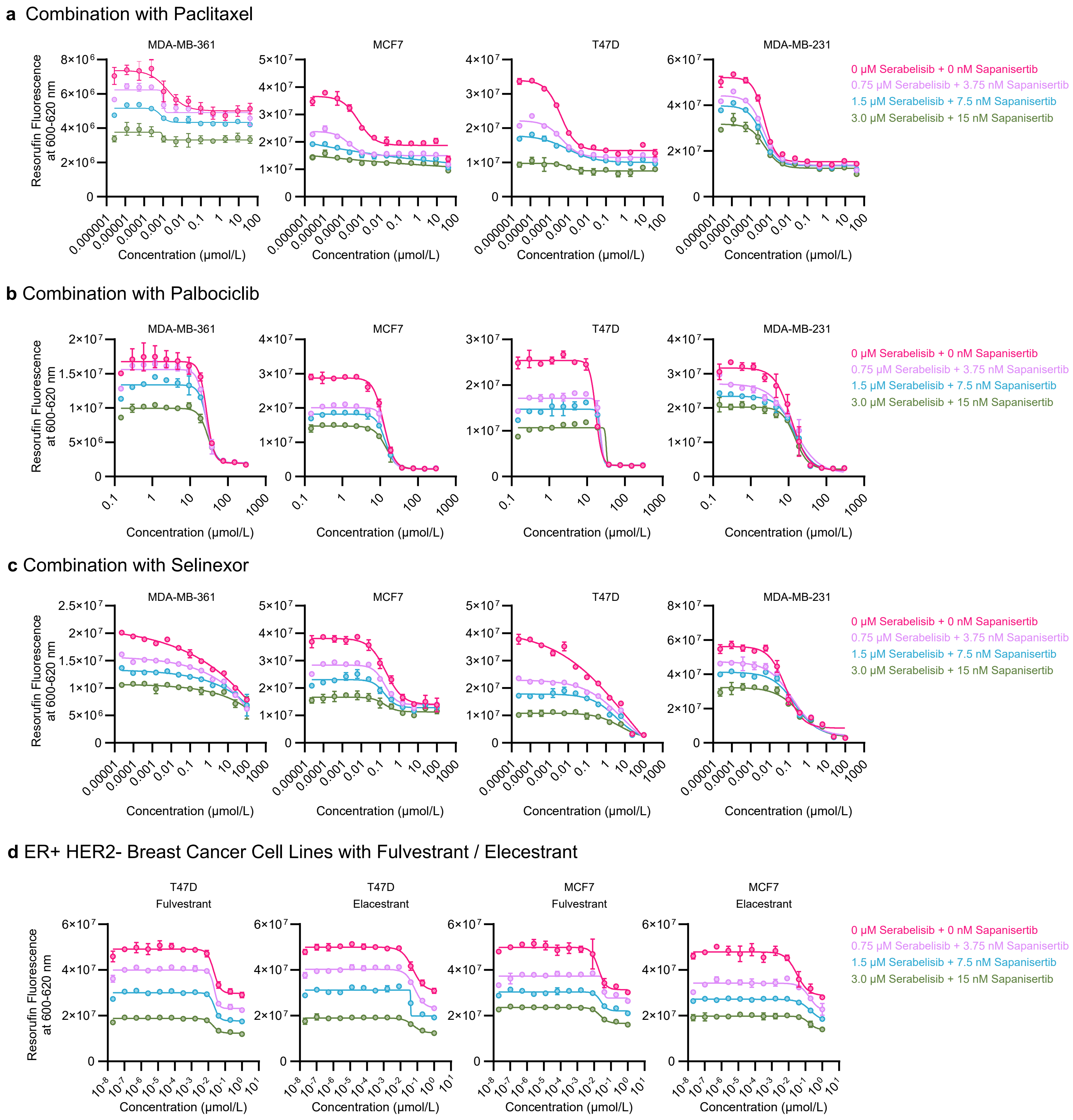

Supplementary Figure 3: Serabelisib and sapanisertib improve breast cancer cell growth-inhibition in combination with a range of targeted and chemotherapeutic anticancer agents. a. Dose-response curves for paclitaxel with different concentrations of serabelisib + sapanisertib at 72 hours post-treatment. **b.** Dose-response curves for palbociclib with different concentrations of serabelisib + sapanisertib at 72 hours post-treatment. **c.** Dose-response curves for selinexor with different concentrations of serabelisib + sapanisertib at 72 hours post-treatment. **d.** Dose-response curves for fulvestrant or elacestrant in ER^+ $HER2^-$ breast cancer cell lines in the presence of 0.1 nM E2, with different concentrations of serabelisib + sapanisertib at 72 hours post-treatment. Error bars denote SD.

Supplementary Figure 4

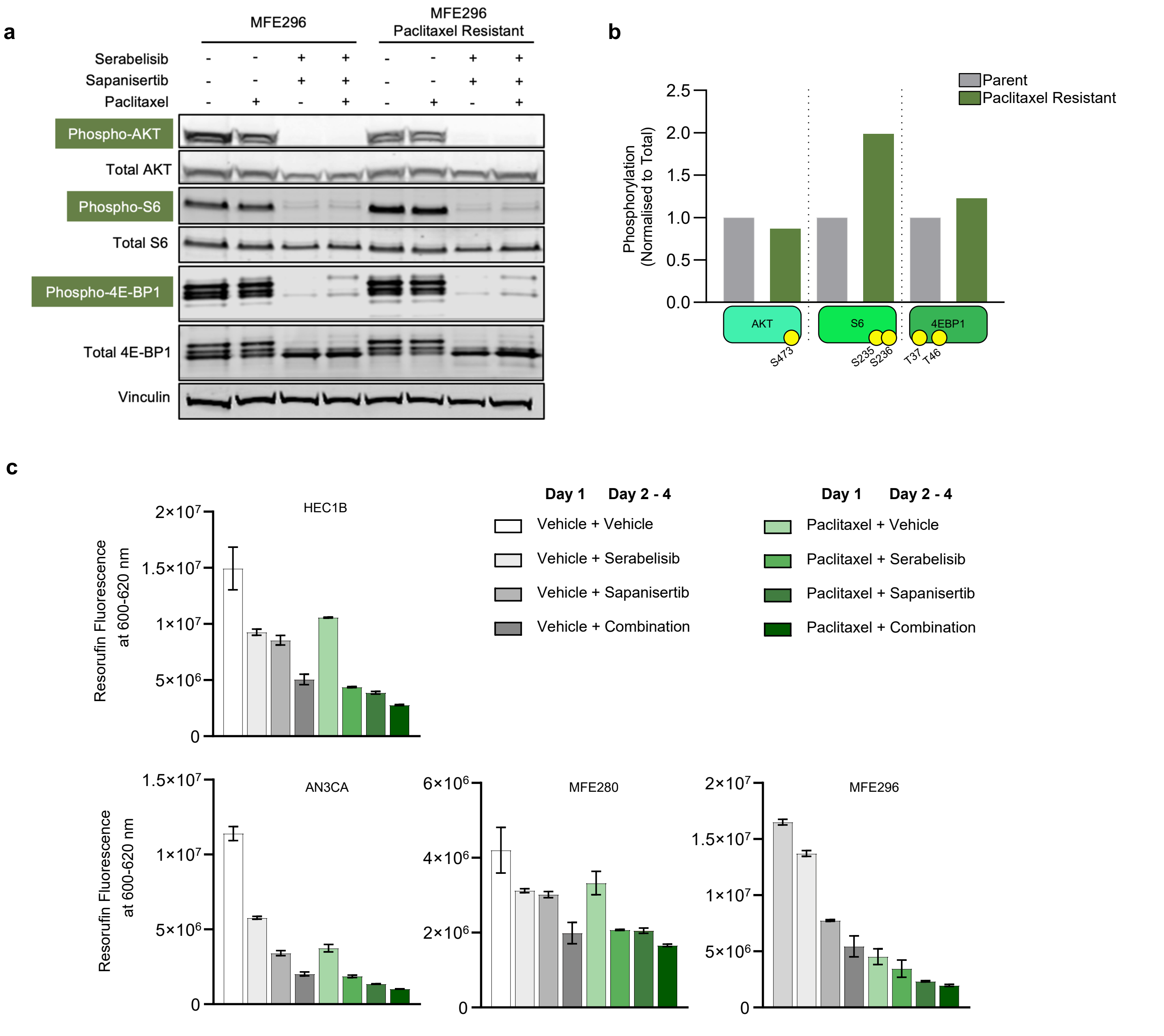

Supplementary Figure 4: Taxane resistance activates the PI3K/AKT/mTOR pathway and serabelisib plus sapanisertib maintains efficacy in paclitaxel resistance . a. Western blots of PI3K/AKT/mTOR pathway activity (phosAKT-S473, phosS6-S235/236 and phos4EBP1-T37/46) in MFE296 parental or paclitaxel resistant cell lines, stimulated with 10 ng/ml insulin followed by treatment with the indicated inhibitors for 3-4 hours. **b.** Quantification of the phospho-AKT/S6/4EBP1 levels in vehicle-treated MFE296 and MFE296 paclitaxel resistant samples. **c.** Serabelisib, sapanisertib and paclitaxel is highly effective when given in a clinically relevant dosing schedule. Cells were treated with 10 nM paclitaxel or vehicle on day 1. After 24 h (day 2), media was replaced, followed by the addition of 5.9 µM serabelisib, 30 nM sapanisertib or 5.9 µM serabelisib + 30 nM sapanisertib until day 4. Error bars denote SD.

Supplementary Figure 5

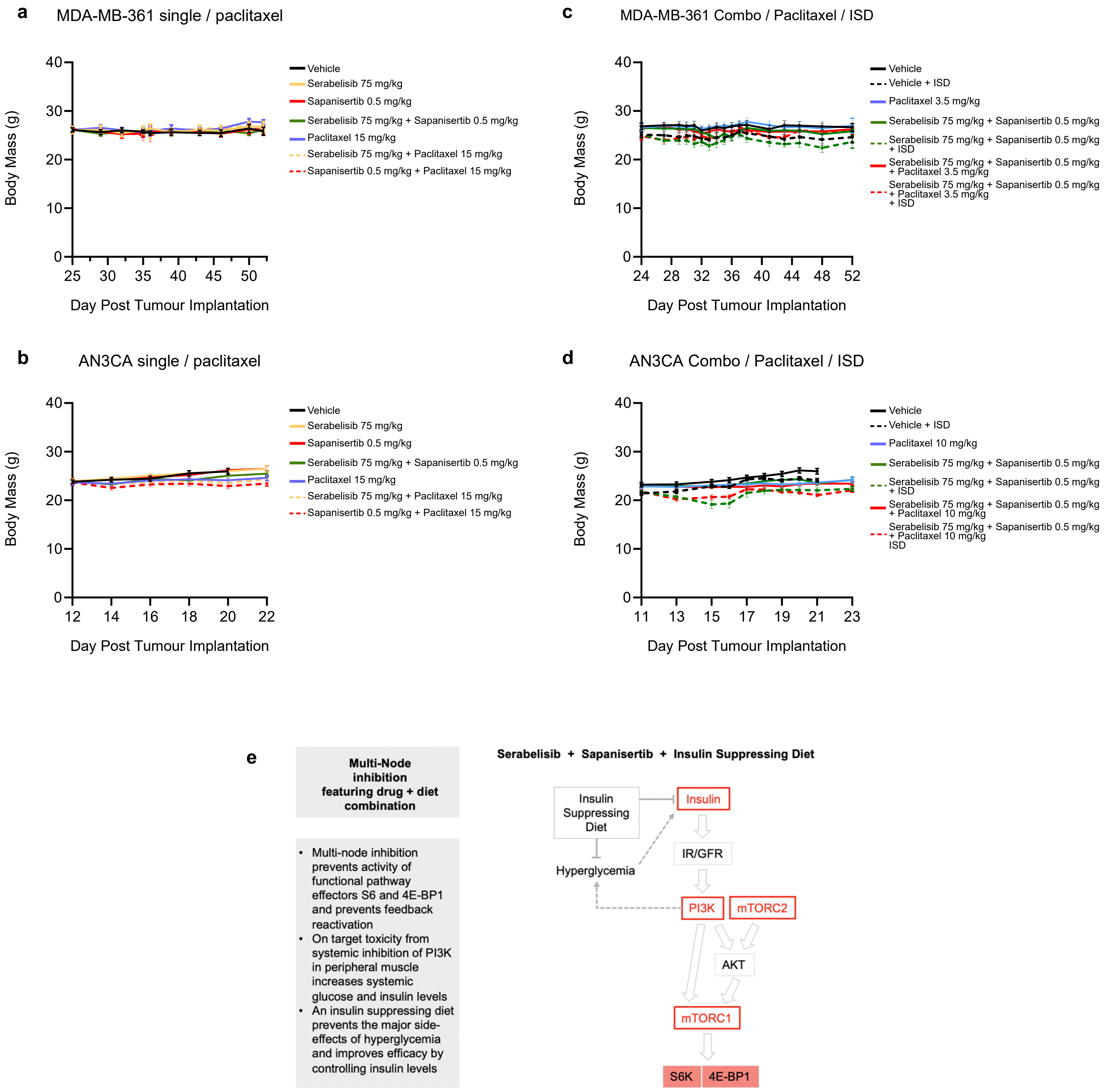

Supplementary figure S5: Serabelisib and sapanisertib in combination with paclitaxel and an insulin suppressing diet is effective and well tolerated. a. Body weight of MDA-MB-361tumour-bearing mice treated with indicated drugs and doses. **b.** Body weight of AN3CA tumour-bearing mice treated with indicated drugs and doses. **c.** Body weight of MDA-MB-361 tumour-bearing mice on chow or ISD diets, treated with indicated drugs and doses. **d.** Body weight of AN3CA tumour-bearing mice on chow or ISD diets, treated with indicated drugs and doses. **e.** Diagram outlining rational combination of multi-node inhibition in combination ISD. n = 5-8 mice per group. Error bars denote SEM.
